# The Editor's Letter-Box

**Published:** 1913-02-22

**Authors:** Lionel S. Lewis

**Affiliations:** St. Mark's Vicarage, Whitechapel, E.


Sir,—I am sure that your sense of fairness will permit this reply to your remarks about the recent special meeting of the constituents of this Fund.

I entirely deny that the holding of the annual meeting in some month other than December could possibly deprive the constituents of the power of electing the Council.

Not withdrawing my motions because a committee had been appointed to consider by-laws was no oversight on my part. As I explained, we want a new suit, but while waiting for it, I tried to put a patch, as I objected to running about ragged and half naked. I have more than once tried to come to terms with the Council of this Fund, but, as you have pointed out, have been put off with technicalities, etc. Therefore, as I held a brief for the sick poor, and the rights of the constituents, and was not trying to win glory for myself and score off my opponents, I decided to pay no attention to the announcement sprung upon me in the room, much as I welcomed it. I felt that a friendly Council which meant to deal fairly with one would have notified me beforehand. . . . The real points before the meeting on the 10th instant, and my resolutions were moved:

(*a*) To give the constituents power to appoint the Distribution Committee.

(*b*) To create machinery to refer a grant to arbitration.

(*c*) To stop the insult of our being called in December to approve the irrevocable acts of August.

And what happened? The spokesman of the Council made an incendiary speech attacking anti-vivisection and anti-vivisectionists, which was absolutely ungermane to the question.

I happen to be an anti-vivisectionist (I am also an antiquary and a few other things). I do not suppose there were ten such in a room of several hundred people. But the meeting was hounded on to shout down all my supporters (were it not for the Lord Mayor's courtesy and firmness I should have shared the same fate), and in a panic the constituents voted to remain in their powerless state, and to endorse this grave state of muddle and scandal which you have denounced. And why? For fear of one anti-vivisectionist. They voted themselves untrustworthy for fear they should at some future date be coerced and cajoled by a handful of people whom they dislike. (Incidentally such craven fear is a triumph for anti-vivisection.)

I claim that no dramatic withdrawal on my part, and no patting myself on the back, would have so shown the rightness of my cause and the need of reform and solid thinking as this result of the debate. I claim that I should have been false to the sick poor and my duty as a constituent had I not tried to put an immediate end to the scandals in question, which may or may not be accomplished by the amendments to the Constitution to be brought before us at some future date.

P.S.—Your memory fails in saying that my agitation was on behalf of the Anti-Vivisection Hospital. As the result of the efforts of many on its behalf that hospital had a grant made to it, which it, in my opinion, unwisely and ungraciously refused owing to some remarks better left unsaid by a private individual at the time of the grant. I disagree with the hospital's action. I, therefore, had not got its case in the dimmest recesses of my mind.

It is equally inaccurate to refer to Mr. Stephen Coleridge as a supporter of that hospital as it is to refer to me as a follower of Mr. Coleridge. I admire his abilities and his humanity, but I often neither agree nor follow. Mr. Coleridge must have smiled. If I be his "mouthpiece," as Mr. Holland also says, then must poor Mr. Coleridge sometimes suffer from hemi-aphasia, for the mouth sometimes speaks what he does not desire.—Yours faithfully,

Lionel S. Lewis.

St. Mark's Vicarage, Whitechapel, E.
February 18, 1913.

* * [Mr. Lewis should read our criticisms more carefully, for he misquotes or misapplies several. Our article in The Hospital of the 15th instant has not the meaning applied to it by Mr. Lewis in the second paragraph of his letter. The fact we pointed out was that the election of the Distribution Committee is vested in the Council; that the Council is elected by the constituents at the December meeting; and thus under No. 1 of the present regulations the constituents indirectly have the power to influence the choice of the members of the Distribution Committee for each year. Again, the announcement of the appointment by the Council of a sub-committee to consider by-laws could not have "been sprung upon" Mr. Lewis in the room, for this decision of the Council was arrived at on January 29, and was published in the daily papers of January 30. It also appeared in THE HOSPITAL, p. 508, on February 8. Pressure on our space has necessitated our omitting from the above letter a repetition of points in Mr. Lewis's speech which we reported fully in our issue of the 15th instant.—ED. THE HOSPITAL.]

